# The time course of the transcriptomic response of *Sinorhizobium meliloti *1021 following a shift to acidic pH

**DOI:** 10.1186/1471-2180-9-37

**Published:** 2009-02-15

**Authors:** Christoph Hellweg, Alfred Pühler, Stefan Weidner

**Affiliations:** 1 Lehrstuhl für Genetik, Fakultät für Biologie, Universität Bielefeld, 33594 Bielefeld, Germany; 2 Center for Biotechnology, Universität Bielefeld, 33594 Bielefeld, Germany

## Abstract

**Background:**

The symbiotic soil bacterium *Sinorhizobium meliloti *often has to face low pH in its natural habitats. To identify genes responding to pH stress a global transcriptional analysis of *S. meliloti *strain 1021 following a pH shift from pH 7.0 to pH 5.75 was carried out. In detail, oligo-based whole genome microarrays were used in a time course experiment. The monitoring period covered a time span of about one hour after the pH shift. The obtained microarray data was filtered and grouped by K-means clustering in order to obtain groups of genes behaving similarly concerning their expression levels throughout the time course.

**Results:**

The results display a versatile response of *S. meliloti *1021 represented by distinct expression profiles of subsets of genes with functional relation. The eight generated clusters could be subdivided into a group of four clusters containing genes that were up-regulated and another group of four clusters containing genes that were down-regulated in response to the acidic pH shift. The respective mean expression progression of the four up-regulated clusters could be described as (i) permanently and strong, (ii) permanently and intermediate, (iii) permanently and progressive, and (iv) transiently up-regulated. The expression profile of the four down-regulated clusters could be characterized as (i) permanently, (ii) permanently and progressive, (iii) transiently, and (iv) ultra short down-regulated. Genes coding for proteins with functional relation were mostly cumulated in the same cluster, pointing to a characteristic expression profile for distinct cellular functions. Among the strongest up-regulated genes *lpiA*, *degP1*, *cah*, *exoV *and *exoH *were found. The most striking functional groups responding to the shift to acidic pH were genes of the exopolysaccharide I biosynthesis as well as flagellar and chemotaxis genes. While the genes of the exopolysaccharide I biosynthesis (*exoY*, *exoQ*, *exoW*, *exoV*, *exoT*, *exoH*, *exoK exoL*, *exoO*, *exoN*, *exoP*) were up-regulated, the expression level of the flagellar and chemotaxis genes (*visR*, *motA, flgF, flgB, flgC, fliE, flgG, flgE, flgL, flbT*, *mcpU*) simultaneously decreased in response to acidic pH. Other responding functional groups of genes mainly belonged to nitrogen uptake and metabolism (*amtB*, *nrtB*, *nirB*, *nirD*), methionine metabolism (*metA*, *metF*, *metH*, *metK*, *bmt *and *ahcY*) as well as ion transport systems (*sitABCD*, *phoCD*). It is noteworthy, that several genes coding for hypothetical proteins of unknown function could be identified as up-regulated in response to the pH shift.

**Conclusion:**

It was shown that the short term response to acidic pH stress does not result in a simple induction or repression of genes, but in a sequence of responses varying in their intensity over time. Obviously, the response to acidic pH is not based on a few specific genes, but involves whole sets of genes associated with various cellular functions.

## Background

The symbiotic interaction between rhizobia and leguminous plants plays an important role in global nitrogen fixation. During symbiosis rhizobia colonize the root nodules and induce nodule formation. Rhizobia in turn differentiate into bacteroids and live as endosymbionts inside plant cells. They fix atmospheric nitrogen and provide the fixed nitrogen to the host plant. The efficiency of this symbiosis is constrained by several factors relating to the soil and the rate of nodulation and nitrogen fixation is diminished. The most commonly observed factors are water deficiency, high temperature, high salt content and low pH (for review see [1]). At acidic pH conditions the bacterial partner is limited in survival and persistence and the nodulation efficiency is reduced [2-4]. Another situation where rhizobia are commonly facing a low pH environment is the rhizoplane of their leguminous host plants, where the pH is decreased by protons and organic acids excreted by the plants [5]. Once a symbiosis has been established the symbiosome has been postulated to form an acidic and lytic compartment [6]. Several research groups have been trying to identify pH tolerant strains [3,7] and to reveal the genetic mechanisms enabling those strains to outperform other strains in low pH soils, however up until now the basis of the rhizobial pH tolerance remains unknown.

Since the genome of *S. meliloti *1021 is well characterised [8-11]*S. meliloti *1021 is considered to represent an ideal candidate to analyse its behaviour under environmental conditions. The response to several stresses has been studied for individual genes of *S. meliloti *on a proteomic as well as a transcriptomic scale [12-15]. But the cellular response of *S. meliloti *to acid stress has so far not been investigated on a genome-wide level. pH stress can affect cells in several ways and therefore different responses exist. Acid tolerance in general is a mechanism of the cell to face an unfavourable acidic condition, whereas an adaptive acid tolerance (ATR) is defined as increased tolerance against low pH after growing cells in moderately low pH media [16] (for review see [17]). For rhizobia most studies about genes involving the acid stress response have been conducted with *S. medicae *(formerly classified as *S. meliloti *WSM 419). By using a transposon mutagenesis system [18] a functionally diverse set of pH responsive and acid tolerance related genes could be identified [19]. Gene products required for acid tolerance in *S. medicae *are for example ActP, a CPx heavy metal transporting ATPase [20], and ActA, an apolipoprotein acyl transferase [21]. A gene coding for a regulatory protein known to be required for the acid tolerance in *S. medicae *is *actR *[22]. The encoded response regulator ActR is activated by its corresponding sensor histidine kinase ActS, whose loss also leads to sensitivity to low pH. The *cbbS *gene involved in CO_2 _fixation and the *narB *gene involved in nitrate assimilation as well as the nitrogen fixation regulator genes *fixK *and *nifA *could be identified as target genes for the regulator ActR [23]. Along with the genes required for low pH tolerance some further genes up-regulated by low pH were identified for *S. medicae *[19,24]. Among these was *lpiA*, a gene found to be necessary for the adaptive acid tolerance (ATR). In *Rhizobium tropici*, the bacterial symbiont of *Phaseolus vulgaris*, this gene was also up-regulated by low pH and was found to be necessary for an increased nodulation competitiveness [25].

In this study the transcriptional response of *S. meliloti *strain 1021 following a pH shift from pH 7.0 to pH 5.75 was analysed on a genome wide level. Using whole-genome Sm6kOligo microarrays [15] the expression of *S. meliloti *genes responding to this environmental change was monitored over a period of one hour. The data obtained was filtered and clustered to obtain groups of genes with a similar behaviour.

## Results and Discussion

### Growth analysis of *S. meliloti *1021 cultures exposed to neutral and acidic pH

The aim of this study was to analyse the transcriptional response of *S. meliloti *1021 following a shift from a neutral to an acidic pH. Since adaptation to new environmental conditions means passing through an evolving process of cellular responses until reaching a steady state balance, it was decided to monitor the transcriptional response over a certain period of time. One critical point concerns the correct choice of parameters for the pH shift. The pH stress should be applied to *S. meliloti *in a manner that it is not lethal for the cells, the central metabolism should also not be turned down on a broad scale, otherwise a global stress and not a pH-specific stress response is to be expected. In order to define appropriate experimental conditions for the pH shift, growth tests in Vincent minimal medium were carried out by varying the pH from 5.5 to 7.0 in 0.25 increments. It turned out that *S. meliloti *1021 is not able to grow at pH 5.5 while above pH 6.0 only minor deviations from the growth curve at pH 7.0 occurred (data not shown). At pH 5.75 *S. meliloti *1021 showed a reduced growth rate, but the cell titer counts documented that this pH was not yet lethal (data not shown).

**Figure 1:**
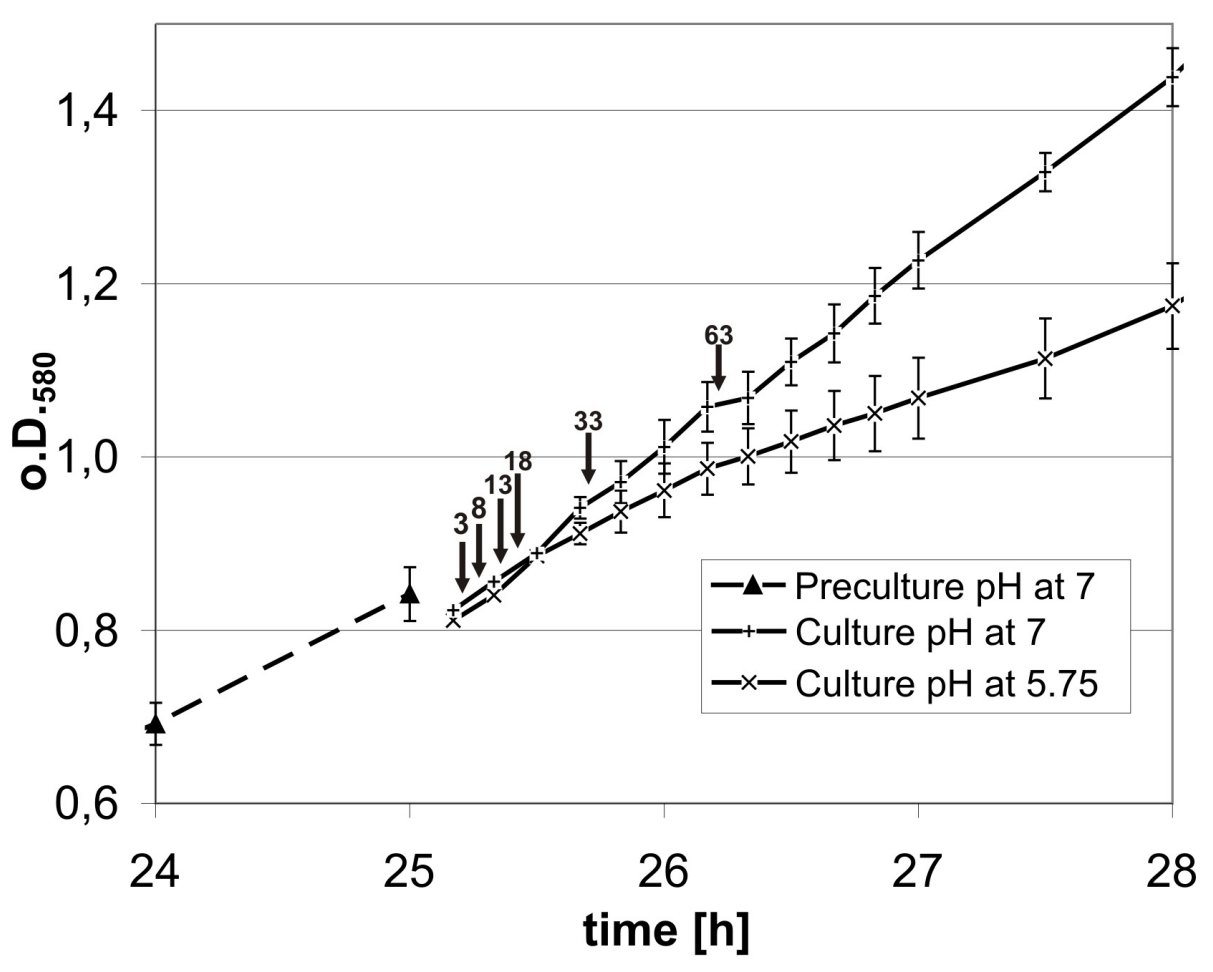
**Growth of *S. meliloti *1021 before and after a shift to low pH**. An *S. meliloti *1021 preculture has been grown in VMM buffered at pH 7.0 until it reached an o.D._580 _of 0.8 (dotted line with triangles). Afterwards the pre-culture has been separated into even parts, centrifuged and re-suspended in VMM at pH 5.75 and VMM at pH 7.0, respectively. The growth of the pH 5.75 culture is given by lines with crosses and the growth of the pH 7.0 culture is given by lines with plus-symbols. The arrows in the diagram indicate the time points where cell culture probes were taken for transcriptional profiling. Remarks indicate the time in minutes passed after the splitting of the *S. meliloti *preculture.

The aim of this study was to identify genes of *S. meliloti *that directly respond to changes of the environmental pH, the transcriptional short term response within the first hour after a pH change was therefore the focus of our interest. In a time course experiment the global gene expression of *S. meliloti *cells exposed to a pH change from 7.0 to 5.75 was compared to the gene expression of untreated cells. To ensure identical conditions and treatment *S. meliloti *1021 cells were grown in VMM at pH 7.0 until an o.D._580 _of 0.8 was reached (Fig. 1), subsequently the culture was split in two and centrifuged. After centrifugation of the split cultures, the used growth medium was decanted and exchanged by fresh VMM adjusted to pH 5.75 (as testing condition) and to pH 7.0 (as reference), respectively. All manipulation steps were carried out very gently by using pre-warmed equipment and material to avoid any unwanted influences on the cells. The growth curves show the effect of the lowered pH on the growth of the *S. meliloti *1021 culture (Fig. 1). The culture that was shifted to pH 5.75 grew slower than the pH 7.0 culture. For the duration of the time course experiment, the pH value of both cultures did not change. At later time points an alkalisation of the growth medium could be observed for the low pH culture (data not shown).

### Cluster analysis of expression profiles of *S. meliloti *genes following a shift to acidic pH

Cells were harvested from both cultures grown at pH 7.0 and pH 5.75 after 3, 8, 13, 18, 33 and 63 minutes (Fig. 1). Because both the sample (pH 5.75) and control (pH 7.0) were obtained from the same starting culture and were treated simultaneously, the differences in the transcriptomic profile should only be a result of the pH shift. For each time point the transcribed and labelled RNA of the pH 5.75 grown culture was hybridised together with the differently labelled RNA of the pH 7.0 reference culture to the Sm6kOligo microarray. The whole procedure was performed in three biological replicates to ensure the validity of the microarray data. The microarray images were analysed using the Imagene Software and EMMA [26] (For microarray data see: http://www.ebi.ac.uk/microarray-as/ae/).

As expected, the microarray analysis for the six successive time points revealed a high number of genes with different expression characteristics over the tested period. In order to identify genes that presumably play a significant role in the cellular response to acidic pH the following filtering criteria were applied. Only genes with a log_2 _fold difference in spot intensities on the microarray slides (M value) of ≥ 2 or ≤ -2 were considered. Because we were also interested in genes that were only transiently active, this limit of significance had to be achieved for at least one time point during the time series. In addition, it was of importance for clustering that each gene was represented with an evaluable expression value (R ≥ 1.5 for both channels) for at least 5 out of the 6 time points. 230 genes fulfilled these filtering criteria. To estimate the number of false positive genes after filtering the false discovery rate (FDR) control was applied for all expression data of these 230 genes. The FDR control revealed a proportion of less than 1% false positives. Additionally, the tendency of the microarray results was confirmed by qRT-PCR for two of the obtained genes (*lpiA *and *phoC*) (data not shown).

Since the *S. meliloti *genome is composed of three replicons with distinctive functional features [8] the distribution of the 230 genes fulfilling the filtering criteria was determined. The percentage of differentially expressed genes of the total number of genes was 3.95% for the chromosome, 2.48% for pSymA, and 4.20% for pSymB. Therefore, compared to the chromosome genes located on pSymB were slightly over represented whereas genes of pSymA were noticeably under represented in the time course experiment. A possible explanation is that pSymA carries mostly symbiosis related genes which are not responding, whereas pSymB and the chromosome contain housekeeping genes. The slight over representation of pSymB might be based on the up-regulation of exopolysaccharide biosynthesis genes (see below).

**Figure 2:**
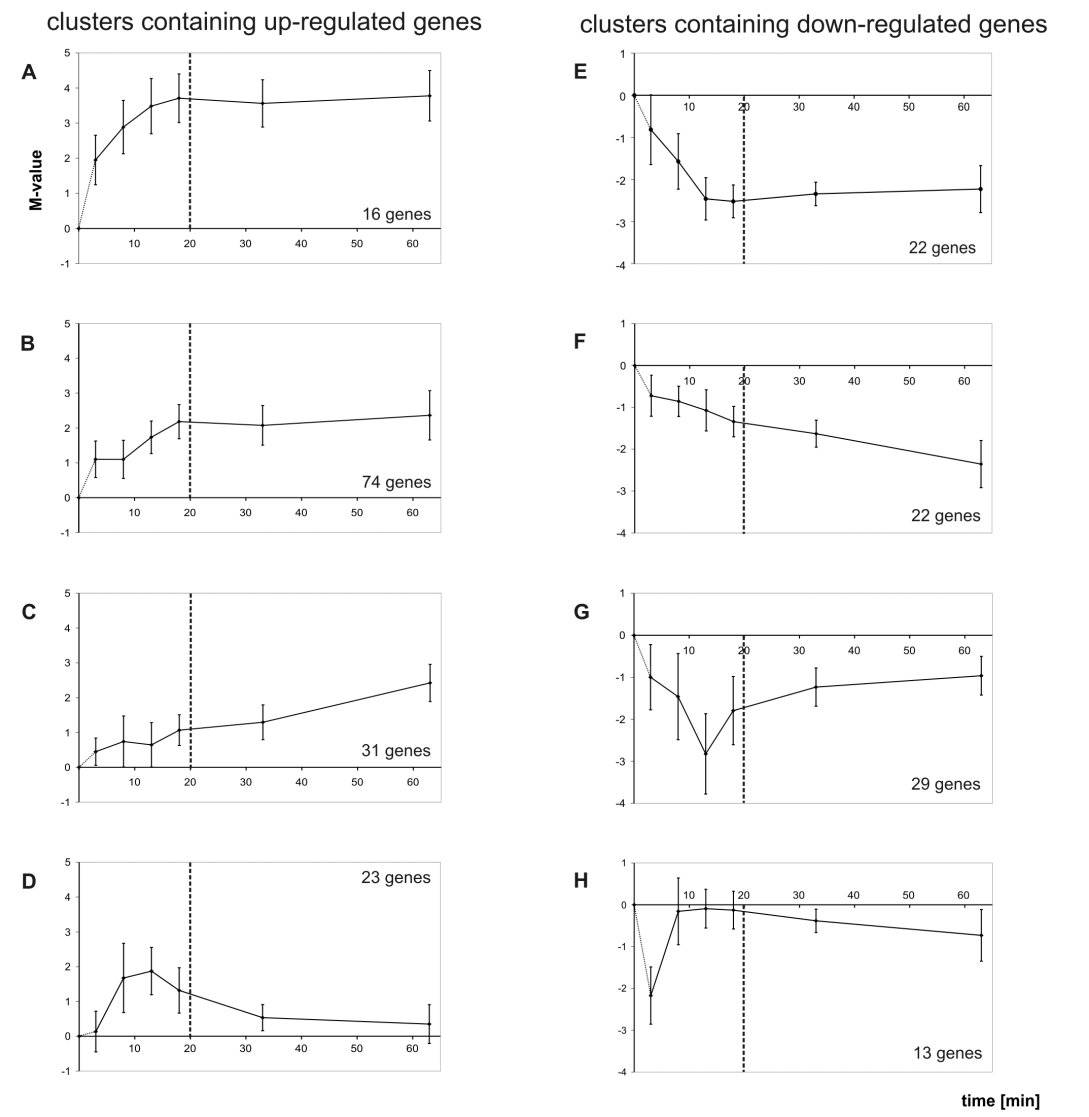
**The eight clusters of the transcriptomic profiling of *S. meliloti *1021 following a shift to acidic pH**. The diagrams of clusters A to H show the mean M value (y-axis) obtained by the Sm6kOligo microarray analyses for each time point (x-axis) after pH shift. The standard deviations are represented by the vertical lines crossing each point of the graph. The dotted line divides the cellular response into two parts. The location of the dotted line was chosen according to the observation that most of the cellular response happened in the first 20 minutes.

In the next step, a clustering of these genes was performed according to their expressional characteristics over time. By hierarchical clustering, a separation into eight different clusters was estimated. Since K-means clustering offers the possibility to group complex time course expression data into a pre selected number of distinct clusters according to their similarity, this method was applied. Eight K-means clusters (see additional files 1, 2, 3, 4, 5, 6, 7 and 8: Heat maps of the generated Clusters A to H; additional file 9: combined spread sheet of the clustered genes) was found to be the smallest number resulting in clusters with clearly distinguishable expression characteristics. These expression characteristics become apparent by calculating the arithmetic mean expression profile of all contained genes (Fig. 2).

**Figure 3:**
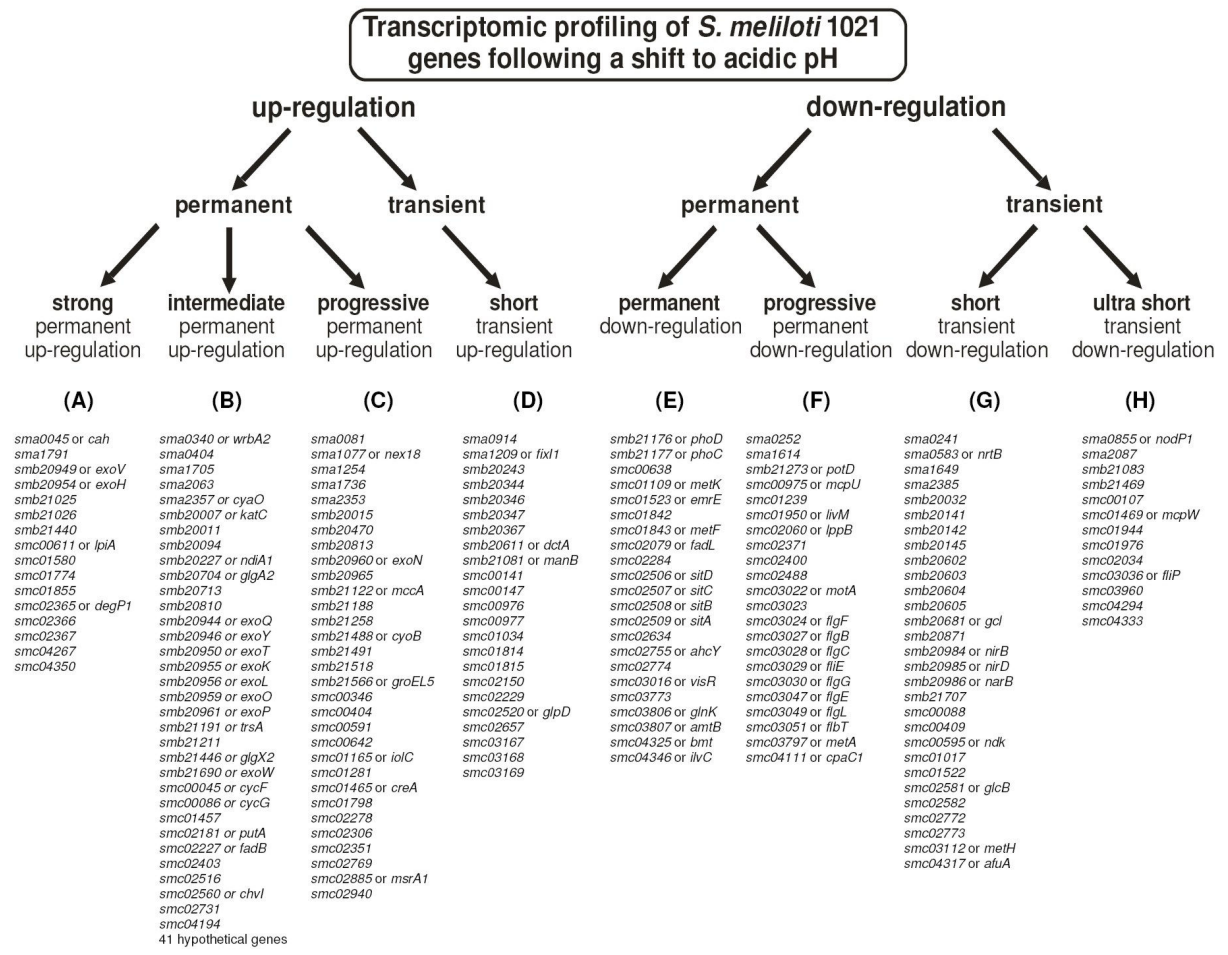
**Grouping of *S. meliloti *1021 genes following a shift to acidic pH**. The eight calculated gene clusters were characterised by their specific transcriptomic response. The figure shows the classification of the genes chosen for clustering by single attributes into the eight clusters calculated by K-means. The tables below give the gene names of the genes distributed to the corresponding clusters.

The cluster analysis generated different groups with discrete expression profiles for up-regulated genes (cluster A to D in Fig. 2) and down-regulated genes (cluster E to H in Fig. 2), not only differing in the intensity of their expression level, but also in their time dependent expression behaviour. These time dependent behaviours can be roughly separated into clusters containing genes that were permanently differentially expressed and clusters containing genes which were only transiently differentially expressed (Fig. 3). An inspection of individual expression profiles (data not shown) indicated that for borderline cases the passage between clusters is fluent. The mean expression profiles of the clusters indicated that the main changes in response to low pH occurred approximately within the first 20 minutes (Fig. 2). After this period of time a constant differential expression level or a constantly changing differential expression level can be observed for most clusters. It is also noticeable that several clusters contain genes organised in operons and groups of genes belonging to related or similar cellular functions.

In cluster A, genes exhibiting a strong and permanent induction accumulated. Genes in this cluster remained up-regulated for the whole observation period. It therefore seems that these genes have a special impact for *S. meliloti *in facing low pH conditions.

Clusters B also contains genes that remained permanently up-regulated in response to the pH shift, but not as strong as those in cluster A.

Cluster C consists of genes that showed a progressive permanent induction in their mean expression behaviour. The expression level of these genes is continuously increasing for the duration of the experiment. Presumably the expression of these genes will reach a plateau phase at later time points.

The mean expression curve of cluster D shows that the genes therein were only transiently up-regulated during the first 10 to 30 minutes following the pH shift. This expressional characteristic suggests that the encoded functions of these genes were only needed for a short period of time.

Cluster E is composed of genes that showed a decrease in their expression up to 20 minutes after the pH shift and thereafter they remained permanently down-regulated.

In contrast, genes contained in cluster F had a progressive permanent repression for the whole duration of the experiment. Similar to cluster C a steady state can be expected for later points of time.

Cluster G consists of genes that were transiently down-regulated in their expression level with a minimum occurring within 20 minutes after the pH shift. In contrast to the genes of cluster D, the encoded functions of the genes in cluster G are likely to be temporarily not needed for the cell.

Cluster H represents the smallest cluster and consists of genes with an ultra short transient repression observed for the first time point of 3 minutes after pH shift.

### Most of cluster A genes encode proteins carrying signal peptides

Cluster A contains 16 genes that showed an increasing induction in response to the pH shift up to 18 minutes and maintained constantly high expression values thereafter (Fig. 2A). It is of special interest that 9 of these 16 genes encode products carrying signal peptides for secretion. This is remarkable since this is by far the highest percentage of putatively secreted proteins for all clusters analysed. It is therefore possible that one of the most immediate and strongest responses of *S. meliloti *to face acidic pH is the secretion of proteins. Five of these genes code for hypothetical proteins (*smb21025*, *smb21026*, *smb21440*, *smc01580 *and *smc01774*). Two of the genes encode for putatively secreted lytic enzymes like a protease (*degP1*) and a putative lysozyme (*smc01855*). An orthologous protein of the *degP1 *gene product could also be identified in *S. medicae *following the growth for 5 days at pH 5.7 [27]. Another interesting gene pair in cluster A namely, *smc02366 *and *smc02367 *coding for a two component system, was found to be located downstream of *degP1*. Whether this gene pair is involved in the transcriptional regulation of *degP1 *has to be investigated. Due to its location next to the highly expressed *degP1 *gene a polar effect influencing the *smc02366*-*smc02367 *expression can not be excluded.

Among cluster A *smc00611 *represents one of the highest up-regulated genes during the time course. An orthologue in *S. medicae *termed *lpiA*, is already well known as a pH responsive gene and it could be shown that it is necessary for the adaptation to low pH [19,28]. For *R. tropici *it was demonstrated that *lpiA *is pH responsive and symbiotically relevant [25]. Recently it was shown that *lpiA *is necessary for the lipid lysyl-phosphatidylglycerol formation in *R. tropici *in low pH minimal media and confers an increased resistance to the cationic peptide polymyxin B [29]. This points to a modification of the exterior cell wall by a change of the lipid-structure. In addition *smc0612 *located downstream of *lpiA *was also found to be highly expressed, but since its expression level was slightly lower it was included in cluster B. The two open reading frames *smc00612 *and *smc00613 *are obviously products of a frameshift mutation of the orthologous gene *acvB *[28] and are therefore probably not functional. It could be shown that a complementation of *S. meliloti *1021 with the *lpiA *and *acvB *genes of *A. tumefaciens *resulted in an enhanced tolerance to acidic pH (C. Sohlenkamp, personal communication).

It has been proposed that a modulated or enhanced lipid biosynthesis, as indicated by the high induction of *lpiA*, can increase the biosynthetic need for bicarbonate [30]. A raised demand for bicarbonate can be associated with the strongly up-regulated expression of *cah*, also found in cluster A. The gene *cah *is coding for a carbonic anhydrase that catalyses the fixation of bicarbonate. Since this gene was also highly up-regulated in response to phosphate starvation of *S. meliloti *it seems not to be specific for low pH stress [15].

Another early induction was observed for *exoV *and *exoH *coding for proteins of the exopolysaccharide I biosynthesis (EPS I). The discussion of this and further genes involved in EPS I biosynthesis will be addressed in a later section.

### The large cluster B contains some exo genes responsible for the biosynthesis of succinoglycan and several *rpoE2 *dependently regulated genes

The expression level of the genes comprising cluster B increased to a medium level in the first 10–20 minutes after the pH shift, and remained at this level until the end of the time course experiment (Fig. 2B).

Cluster B represents the biggest cluster and includes 74 genes. This cluster mainly consists of genes coding for hypothetical or conserved hypothetical proteins (41 genes) predominantly located on pSymA or on the chromosome. For these genes no further functional prediction can be given. Besides these genes, eight *exo *genes were found whose products together with the three *exo *genes grouped in cluster A and C are involved in the synthesis of exopolysaccharide I, also termed succinoglycan. In addition, the gene *chvI *coding for a regulator is part of this cluster. The genes of the EPS I biosynthesis are discussed in more detail in a following section.

The gene *katC *present in cluster B was annotated as a catalase. The induction of a catalase in response to low pH seems reasonable to decompose hydrogen peroxide, since a lowered pH favours the generation of radicals by the Fenton reaction. The gene *katC *is known to be regulated in a heat dependent mechanism by *rpoE2 *in *S. meliloti *1021 [31]. Altogether 15 out of 41 described genes being *rpoE2 *dependent regulated under heat stress [31] were found exclusively in cluster B. This is not only indicating a possible role of RpoE2 in the pH stress response but also a specific expression profile of the target genes. Besides *katC*, *ndiA*, *glgA2 *and *glgX2 *the remaining 11 genes are coding for hypothetical proteins. The *rpoE2 *gene itself was filtered for clustering with maximum log_2 _fold expression values of 1.36 and 1.07 at time points 18 minutes and 33 minutes, respectively.

### Cluster C contains among others genes coding for a chaperone and a component of a low O_2 _affinity oxidase

Cluster C contains 31 genes whose expression continuously increased during the time course experiment (Fig. 2C). With over 50% (16 of 31 genes) this cluster resembles cluster B composed of a large amount of genes coding for hypothetical proteins.

In this cluster *groEL5 *could be found, which was the only differentially expressed gene coding for a chaperone. This gene has recently been shown to be specialised for the *S. meliloti *stress response [32]. Besides the DegP1 protease encoding gene, this is the only quality control system found to be up-regulated after the pH shift. In contrast to *degP1 *the *groEL5 *gene was not immediately up-regulated after the pH shift, but slowly increasing in its expression level during the time course. With *nex18 *a gene with unknown function could be detected, which was already shown to be higher expressed during symbiosis and in response to nutrient deprivation stress [33,34].

The gene *cyoB *of the *cyoABC *operon was also included in cluster C. The operon codes for a cytochrome *o *ubiquinol oxidase, a low O_2 _affinity oxidase with a high proton pumping activity. It is noteworthy that *qxtA*, a gene coding for part of the subunit of a high O_2 _affinity oxidase displayed an expression profile similar to genes of cluster C, but was filtered out for clustering analysis due to missing values for three time points. It is known that an increased ΔpH affects the expression of genes of the oxidative phosphorylation. In *S. medicae *the transcriptional induction of *fixN*, a symbiosis related high O_2_-affinity oxidase with a low proton pumping activity was observed after overnight growth at low pH [19]. For *Brucella abortus *it was demonstrated that an interruption in the orthologue of the *qxtAB *operon, named *cydAB*, caused high acid sensitivity [35]. In *E. coli *the gene expression of the orthologues of the low O_2 _affinity oxidase encoded by *cyoABC *and the *qxtAB *encoded high O_2 _affinity oxidase was dependent of the pH [36] with a preferred expression of the high O_2 _affinity oxidase at low pH. Since both, the *cyoABC *and the *qxtAB *systems of *S. meliloti *have so far not been further investigated, their specific role in the pH response cannot be defined.

### Cluster D comprises carbon uptake and fatty acid degradation genes

The mean expression of the genes grouped into cluster D shows a transient induction during the time course (Fig. 2D). In cluster D the *dctA *gene coding for the DctA dicarboxylate import system was found. The DctA dicarboxylate import system [37] is well characterised and a broad substrate range has been identified [38]. This dicarboxylate import system is known to be essential for symbiosis since it is supposed to provide the cells in the bacteroid state with tricarbonic acid (TCA) cycle intermediates from the host plant, e.g. succinate, malate, and fumarate.

A group of genes in this cluster points to an induced fatty acid degradation. The gene *smc00976 *is coding for a putative enoyl CoA hydratase and *smc00977 *and *smc02229 *are coding for putative acyl CoA dehydrogenase proteins. With *glpD*, a gene coding for a glycerol-3-phosphate dehydrogenase involved in the glycerol degradation could also be found in cluster D. The transient induction of genes involved in fatty acid degradation might be related to a lack of energy or the modification of the membrane lipid composition.

### Cluster E contains genes involved in nitrogen metabolism, ion transport and methionine metabolism

Cluster E consists of 22 genes whose expression was lowered in response to the pH shift. The expression was lowered up to 10 minutes after pH shift and then stayed constant until the end of the time course experiment (Fig. 2E).

Cluster E contains genes involved in nitrogen metabolism. The gene *glnK *codes for a PII nitrogen regulatory protein activated under nitrogen limiting conditions and forms together with *amtB*, which encodes a high affinity ammonium transport system, an operon. The GlnK protein could also be identified as lower expressed after a short exposure of *S. medicae *cells to low pH [27]. It was argued by Reeve *et al*. that this observation might be related to some crosstalk between nitrogen and pH sensing systems during the early pH adaptation [27]. With *metF*, *metK*, *bmt*, and *ahcY *four genes involved in the methionine metabolism were also grouped in this cluster, while two other *met *genes were grouped into cluster F (*metA*) and cluster G (*metH*), respectively. The distribution of these genes to two other clusters of down-regulated genes might be due to the fact the *met *genes are not organised in an operon, but dispersed over the chromosome. S-adenosylmethionine is formed from methionine by MetK and is the major methylation compound of the cell that is needed e.g. for polyamine- or phosphatidylcholine biosynthesis. The connection between the down-regulation of the methionine metabolism and the pH response is not clear. It was shown that various abiotic stresses result in a rapid change of cellular polyamine levels [39-41].

Several genes belonging to ion uptake systems were located in cluster E, like the complete *sitABCD *operon and *phoC *and *phoD *of the *phoCDET *operon. The *sitABCD *operon codes for a manganese/iron transport system [42,43]. Since an acidic pH causes a higher solubility of ions, the repression of metal ion import systems is likely to be a response to their higher availability. The *phoCDET *operon, codes for a high affinity phosphate transport system [44]. A *phoB *dependent control of *phoCDET *could be observed in *S. meliloti *[15] and in *E. coli *it could be shown that *phoB *is involved in the acid shock response [45].

### Cluster F is almost exclusively composed of genes playing a role in chemotaxis and motility

Cluster F consists of genes whose expression was continuously lowered during the time course experiment (Fig. 2F). It mainly comprises genes (10 of 22) belonging to chemotaxis and flagellar biosynthesis (*flgB*, *flgG*, *flgL*, *flgF*, *flgC*, *flgE*, *fliE*, *flbT*, *motA*, *mcpU*). This phenomenon will be discussed in more detail later.

Another gene in cluster F was *lppB *coding for a lipoprotein, which is a major outer membrane component was grouped in cluster F. A similar expression profile as those of the flagellar biosynthesis and chemotaxis genes confirms a possible co-regulation as was observed in *Salmonella enterica *[46].

### Cluster G consists of several genes involved in nitrogen uptake and utilization

Cluster G consists of genes whose expression was transiently lowered between 8 and 18 minutes following the pH shift and afterwards returned nearly to the ground state (Fig. 2G).

The genes *nirB*, *nirD *and *narB *were distributed to this cluster and are coding for nitrite and nitrate reductases forming ammonia from nitrate. A homologue of *narB *was found to be regulated by the low pH and microaerobiosis regulator ActR in *S. medicae*. A gene coding for an element of a nitrate import system (*nrtB*) could also be found in this cluster, while the remaining two elements of this system encoded by *nrtA *and *nrtC *were not included in the analysis because their expression values were below the threshold for filtering.

Additionally, genes coding for an ABC transport system (*smb21707*, *smb20602*, *smb20603*, *smb20604 *and *smb20605*) sharing homologies with amino acid and urea/short chain amide transport systems are present in cluster G. In addition to this transport system genes (*smb20141*, *smb20142*) of an ABC transport system homologous to the Dpp system from *E. coli *were also grouped in this cluster. This system is known for the import of dipeptides to provide the cell with essential amino acids, nitrogen and energy [47].

### Cluster H is formed by genes with distinct biological functions and a high variation in their expression levels

Cluster H consists of 13 genes that were transiently lower expressed on the very beginning of the time course (Fig. 2H). The proposed encoded functions of the genes in this cluster were found to be very diverse. A secreted peroxidase gene (*sma1944*) [48], a flagellar biosynthesis gene (*fliP*), a chemotaxis sensory gene (*mcpW*), a nodulation gene (*nodP1*) and several hypothetical protein encoding genes were identified to be in cluster H. Interestingly, *nodP *was described as up-regulated at acidic pH in *Rhizobium tropici *[49], but in the study of Moron *et al*. cells were grown for at least one day in low pH media.

### The time resolved expression profile of the *S. meliloti *1021 exo genes and flagellar genes following a shift to acidic pH

Overall the number of differentially expressed genes belonging to the group of EPS I biosynthesis genes and to the group of genes involved in flagellar biosynthesis and motility is striking. Most *exo *genes were joined together in cluster B whereas most flagellar genes were grouped together in cluster F. Furthermore, it is noticeable that the expression of the two groups of genes displayed oppositional characteristics. The EPS I biosynthesis genes responded with a fast then constant induction for the duration of the time course, whereas the flagellar genes were increasingly down-regulated. For *A. tumefaciens *a similar response in succession to pH stress could be identified [50]. In case of *A. tumefaciens *the transcriptome profiling was performed after 7 hours of growth in low pH. Also in our experiment the expressional characteristics of the *exo *and flagellar genes indicated that their response to acidic pH conditions lasts longer than the monitored period of one hour.

**Figure 4:**
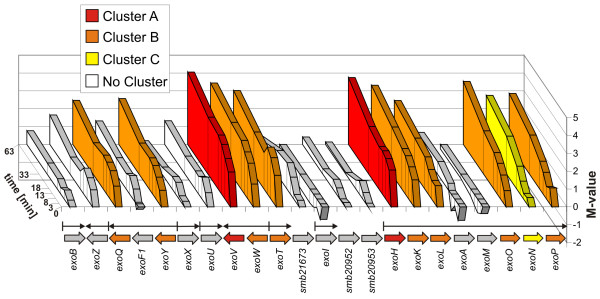
**Map of genes in the EPS I biosynthesis region on pSymB and their expression in response to acidic pH**. The EPS I biosynthesis gene region on pSymB is schematically displayed with its genes given by open arrows coloured according to the K-means cluster distribution. Gene names are given below. Black arrows indicate known operon structures in this region. The graph above shows on the Y-axis the time after pH-shift and on the Z-axis for each time point the expression of the corresponding genes by the M value.

The regulator coding gene *chvI *was with most of the *exo *genes distributed to cluster B. Like in *A. tumefaciens *the gene *chvI *was up-regulated together with several genes responsible for the succinoglycan biosynthesis [50], although it is believed that *chvI *is a negative regulator of the *exo *genes [51]. A closer view on the individual expression levels of the genes of the EPS I biosynthesis gene cluster on pSymB during the time course (Fig. 4) reveals the high induction levels for the majority of the *exo *genes. The maximum induction in the observation period was always reached at 63 minutes after pH shift. Besides the eight *exo *genes found in cluster B, three *exo *genes grouped in cluster A and C. The *exo *genes in cluster A (*exoV *and *exoH*) were among the strongest up-regulated genes in this experiment. The products of these genes are responsible for the final steps of the EPS I biosynthesis. They are involved in the succinylation and pyruvilation of EPS I. It could already be shown for *S. meliloti *that a mutant strain of *exoH *is sensitive to low pH [52], indicating a particular impact of *exoH *on the pH tolerance and of the EPS I biosynthesis genes on the pH tolerance in general. The higher expression value of *exoH *compared to other *exo *genes might also be caused by its position as the first gene in a large operon (*exoHKLAMONP*) [53]. The central genes of this operon (*exoA *and *exoM*) did not show a significant change in their expression level during the time course in contrast to the bordering genes. This might be caused by mRNA instability and degradation effects. Three predicted open reading frames of unknown function within the *exo *gene region (*smb21673*, *smb20952 *and *smb20953*) did not show a differential expression during the time course indicating that they are not protein coding or that they are not connected to the EPS I biosynthesis. Overall the observed induction of *exo *genes is in agreement with the mucoid phenotype observed for *S. meliloti *after growing on low pH plates (data not shown). In low pH soils this response could be a strategy of the cell to establish a more favourable microenvironment by secreting succinoglycan. It was shown that an EPS I overproduction results in a reduced nodulation efficiency [54], therefore the induction of EPS I biosynthesis genes could also be one of the reasons for the observed limited nodulation efficiency of rhizobia in low pH soils [2].

**Figure 5:**
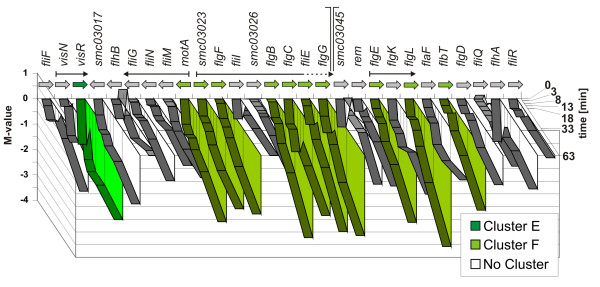
**Map of genes of the flagellar biosynthesis region on the chromosome of *S. meliloti *1021 and their expression in response to acidic pH**. A part of the flagellar gene region is schematically displayed with its genes given by open arrows coloured according to the K-means cluster distribution. Gene names are given below. Black arrows indicate known operon structures. The graph above shows on the Y-axis the time after pH-shift and on the Z-axis for each time point the expression of the corresponding genes by the M-value. For clarity a region of 13 consecutive genes of the flagellar operon (*flgA *– *fliK*) has been omitted. The location of the omitted region is indicated by the orthogonal lines. The ending of a flagellar operon within the omitted region is depicted by a dotted black arrow.

Whereas the *exo *gene expression was increased, several genes of chemotaxis and flagellar biosynthesis (*flgB*, *flgG*, *flgL*, *flgF*, *flgC*, *flgE*, *fliE*, *flbT*, *motA*, *mcpU*) were decreased in their expression levels. After 63 minutes of low pH treatment the genes have reached the highest level of repression. VisR is the main activator of the flagellar genes and forms together with VisN the top layer of a hierarchy of three expression classes. Since the *visN *gene expression was decreased early in the time course experiment (therefore *visN *was grouped into cluster E) the other flagellar genes follow the repression of their activator [55]. The gene coding for the subordinated regulator Rem [56] was also decreasingly expressed with time, but did not reach the threshold for clustering. A detailed consideration of the expression levels of the flagellar biosynthesis genes on the chromosome (Fig. 5) reveals a repression of the complete region, with some parts responding stronger than others. The decreased expression level of *motA*, *flgF *and *flgE *is likely to be a result of their first position in an operon. It is noticeable that among the 10 down-regulated and strongly responding flagellar genes in cluster F five are coding for parts of the rod (*flgF*, *flgB*, *flgC*, *fliE *and *flgG*) and two for parts of the hook (*flgE *and *flgL*) of the flagellum. The genes *motA*, *fliM*, *fliN *and *fliG *are proposed to form an operon [55]. While the expression of *motA*, which is coding for a transmembrane proton channel protein, was decreased in the time course experiment, the other three genes which encode flagellar switch proteins did not respond to the shift to acidic pH. If this behaviour is caused by a specific regulation or is due to mRNA degradation processes cannot be answered. It is known that the assembly of the flagellar apparatus follows a strict sequence of steps beginning with the MS- and C- rings followed by the export apparatus, rod, hook and ending with the flagellar filament and energizing complexes [57]. The results presented here indicate that the disassembly is also performed in a defined order. The loss of flagellar motility at low pH could already be shown for the closely related *Rhizobium leguminosarum *bv.*viciae *and *A. tumefaciens *[50,58], whereas the more distantly related enterobacteria *E. coli *and *Salmonella enterica *serovar Thyphimurium showed an opposite response [59-61]. For cases of induced motility it was argued that at low pH the large ΔpH drives flagellar rotation [62]. Since there are also reports of *E coli *where it could be demonstrated that motility is lost at low pH [63] the picture is ambiguous. A turndown of the flagellar motility genes of *S. meliloti *was also observed for other stresses like osmotic stress [14,64], heat shock and nutrient starvation [31]. It is therefore apparent that this response is a general stress response of *S. meliloti *1021 and not an answer specific for pH stress. Since cell motility is very energy consumptive, the repression of the motility genes is likely to save energy which is needed to face the low pH e.g. by enhancing the EPS I biosynthesis.

## Conclusion

This study demonstrates the complexity of the cellular response of *S. meliloti *to adapt to a new environmental conditions. The mechanism of the cell to face the low pH is a mixture of several distinct reactions which follow a particular order in time. By applying K-means clustering analysis the diversity of different responses of individual genes was reduced to 8 main expression profiles. By this method a reasonable distinction between differently behaving up-regulated and down-regulated genes could be performed. Furthermore, within the obtained clusters, groups of genes with functional relationship were often joined together. Additionally, this analysis revealed that within the first 20 minutes after the shift to acidic pH the cell appears to perform the main changes necessary to adapt to the new environmental circumstances on the transcriptional level.

The immediate response of *S. meliloti *to low pH in the main appears to be the modification of the cell surface, since several among the highest up-regulated genes (cluster A) encode for probably secreted proteins. The differential expression of some genes was obviously only of temporary need for the cell until about 20 minutes after pH shift (as indicated by clusters D and G). Possibly an increasing demand for energy causes the activation of the dicarboxylate transport system gene *dctA *and of several genes of the fatty acid degradation (cluster D) while at the same time genes for nitrogen uptake and utilization (cluster G) and amino acid biosynthesis were lower expressed. The latter was clearly indicated by the lowered expression of several methionine metabolism genes.

Several genes contributing to the EPS I biosynthesis were up-regulated in response to the acidic pH shift. The secretion of EPS I might be an attempt of the cell to ameliorate the environment. In parallel a decreasing expression of motility genes can be regarded as an attempt of the cell to save energy.

The transcriptional response of *S. meliloti *1021 towards low pH showed several parallels to the response in *A. tumefaciens *[50], with the induction of the *exo *genes and the repression of motility genes. Mechanisms to actively compete against a lowered pH like e.g. in *E. coli *by decarboxylation of amino acids (for review see [65])[66] could not be identified. Possibly in oligotrophic soils a metabolisation of amino acids is inappropriate.

Overall this work showed that the short term response to acidic pH stress does not result in a simple induction or repression of genes, but in a sequence of responses varying in their intensity over time. This indicates that a comprehensive analysis of the transcriptional response of a cell confronted with a new environmental situation requires a monitoring over a longer period of time and not only the analysis of a snap shot. Obviously, the response to acidic pH is not based on a few specific genes, but involves several genes associated with various cellular functions. On the other hand, a considerable part of the responding genes belongs to the group of hypothetical genes. These genes represent promising objectives for future investigations.

## Methods

### Media and growth conditions

*S. meliloti *strain 1021 was cultivated in Erlenmeyer flasks at 30°C in Vincent minimal medium (VMM) [67] and shaken at 140 rpm. With exception of 37 μM iron(III) choride no additional metals have been added to the VMM. The pH of the VMM was adjusted by using either HCl or NaOH. Precultures were grown in tryptone yeast complex medium [68] with appropriate antibiotics (600 μg/ml streptomycin).

For pH shift experiments cells of three independent cultures were grown in 100 ml buffered VMM (20 mM BisTris) to an o.D._580 _of 0.8. All of the following steps were carried out under gentle conditions using pre-warmed equipment. Cell cultures of each flask were halved in two even parts of 50 ml, centrifuged (10000 × g, 2 min, 30°C) and the supernatant was discarded. The cell pellets were resuspended in 50 ml VMM with pH 5.75 and 50 ml VMM with pH 7.0, respectively, and incubated at 30°C. At six time points cell suspension probes of 5 ml were harvested from each flask. Immediately centrifuged (10000 × g, 1 min, 4°C) the resulting pellets were instantly frozen by liquid nitrogen for later RNA preparation. Cell suspension probes were harvested at 3, 8, 13, 18, 33, and 63 minutes following the pH shift.

### RNA isolation

RNA was isolated according to the protocol published by Rüberg *et al*. [14]. Total RNA was prepared using the RNeasy mini kit (QIAGEN, Hildesheim, Germany). By ribolysation (30 s; speed, 6.5; Hybaid, Heidelberg, Germany) cells were disrupted in the RLT buffer provided with the kit in Fast Protein Tubes (Qbiogene, Carlsbad, CA).

### Transcriptional profiling using the SM6kOligo whole genome microarray

The well established Sm6kOligo microarray described by Krol and Becker [15] was employed for transcriptional profiling. For each preparation of Cy3 and Cy5 labelled cDNAs 10 μg of total RNA were used [69]. To each microarray the cDNA of the pH 7.0 and pH 5.75 grown cultures were mixed and hybridised. The microarray experiments were performed in three biological replicates.

The acquisition of the microarray images was performed as described previously [14,15]. By using the ImaGene 5.0 software (Biodiscovery Inc., Los Angeles, CA, USA) the mean signal and mean local background intensities for each spot were identified and calculated. If R was ≤ 1.5 in both channels, spots were flagged as "empty", the remaining spots were used for further analysis. The log_2 _value of the intensity ratios (M_i_) was calculated for each spot with M_i _= log_2_(R_i_/G_i_). R_i _= I_ch1(i)_-Bg_ch1(i) _and G_i _= I_ch2(i)_-Bg_ch2(i) _with I_ch1i _and I_ch2i _being the intensities of a spot in channel l or channel 2 and Bg_ch1(i) _and Bg_ch2(i) _being the background intensity of a spot in channel 1 or channel 2, respectively. The mean intensity was calculated for each spot with A_i _= log_2_(R_i_G_i_)^0.5^. Normalization and t-statistics were carried out using the Emma 1.1 microarray data analysis software [26]. It should be mentioned that in this work genes with a positive M value are addressed as "up-regulated" and genes with a negative M value are addressed as "down-regulated", although a positive value will also be calculated if a gene is less strong down-regulated under pH 5.75 than under pH 7.0 and vice versa.

The microarray results were verified for specific genes (*lpiA *and *phoC*) by quantitative reverse transcription-PCR using a QuantiTect SYBR Green reverse transcription-PCR kit (QIAGEN, Hildesheim, Germany) according to the manufacturer's instructions.

### Filtering and clustering analysis of the microarray data

For clustering purposes only those genes were taken into account which had an evaluable expression value for at least 5 of 6 time points and for which at least one time point had an M value of ≥ 2 or ≤ 2. Expression values are evaluable if the value for R is ≥ 1.5 for both channels. 230 genes fulfilled these criteria. For these 230 genes 444 time points showed an M value of ≥ 2 or ≤ -2. In testing these time points for an FDR (False Discovery Rate) corrected P value of ≥ 0.05, only 4 results (≈ 0.9%) were above this value. These were: t3 *smc01523 *P = 0.07, t33 *smc04173 *P = 0.09, t63 *smb21026 *P = 0.06, and t63 *sma1736 *P = 0.22. For K means clustering analysis of the microarray experiment data the Genesis software was used (Sturn, 2001; http://genome.tugraz.at/genesisclient/genesisclient_description.shtml). The K means clustering was carried out in 8 groups.

## Authors' contributions

CH and SW designed the study, CH performed all works. SW and AP provided critical expertise for the manuscript. All authors read and approved the final manuscript.

## Supplementary Material

Additional file 1 **Heat map of cluster A. By K-means the transcriptional data obtained by microarray analysis of the *S. meliloti *1021 pH shock time course experiment were grouped into eight clusters.** In cluster A, genes exhibiting a strong and permanent induction were accumulated. Genes in this cluster remained up-regulated for the whole observation period. Presumably, these genes have a special impact for *S. meliloti *in facing low pH conditions. Each column of the heat map represents one time point after shift from pH 7.0 to pH 5.75 in the following order: 3, 8, 13, 18, 33, and 63 minutes. The values in the boxes are the M-values of a specific gene represented in a row. The background colour visualises the strength of the induction/lower expression (red/green) by the colour intensity. Click here for file

Additional file 2 **Heat map of cluster B of the eight clusters calculated by K-means clustering of the transcriptional data obtained by microarray analysis of the *S. meliloti *1021 pH shock time course experiment.** Cluster B is the largest cluster. The genes in this cluster are permanently up-regulated in response to the pH shift. It contains *exo *genes responsible for the biosynthesis of succinoglycan and several genes which are *rpoE2 *dependently regulated. Among the genes in cluster B several encode for hypothetical proteins. Each column of the heat map represents one time point after shift from pH 7.0 to pH 5.75 in the following order: 3, 8, 13, 18, 33, and 63 minutes. The values in the boxes are the M-values of a specific gene represented in a row. The background colour visualises the strength of the induction/lower expression (red/green) by the colour intensity. Click here for file

Additional file 3 **Heat map of cluster C of the eight clusters calculated by K-means clustering of the transcriptional data obtained by microarray analysis of the *S. meliloti *1021 pH shock time course experiment.** Cluster C contains over 50% hypothetical genes and a gene coding for a chaperone as well as for a component of a low O_2 _affinity oxidase. The genes in cluster C showed a progressive permanent induction in their mean expression behaviour. Each column of the heat map represents one time point after shift from pH 7.0 to pH 5.75 in the following order: 3, 8, 13, 18, 33, and 63 minutes. The values in the boxes are the M-values of a specific gene represented in a row. The background colour visualises the strength of the induction/lower expression (red/green) by the colour intensity. Click here for file

Additional file 4 **Heat map of cluster D of the eight clusters calculated by K-means clustering of the transcriptional data obtained by microarray analysis of the *S. meliloti *1021 pH shock time course experiment.** Cluster D comprises carbon uptake and fatty acid degradation genes. The containing genes were transiently up-regulated during the first 10 to 30 minutes following the pH shift. Each column of the heat map represents one time point after shift from pH 7.0 to pH 5.75 in the following order: 3, 8, 13, 18, 33, and 63 minutes. The values in the boxes are the M-values of a specific gene represented in a row. The background colour visualises the strength of the induction/lower expression (red/green) by the colour intensity. Click here for file

Additional file 5 **Heat map of cluster E of the eight clusters calculated by K-means clustering of the transcriptional data obtained by microarray analysis of the *S. meliloti *1021 pH shock time course experiment.** Cluster E contains genes involved in nitrogen metabolism, ion transport and amino acid biosynthesis. These genes were decreased in their expression value up to 20 minutes after pH shift and then stayed permanently down-regulated. Each column of the heat map represents one time point after shift from pH 7.0 to pH 5.75 in the following order: 3, 8, 13, 18, 33, and 63 minutes. The values in the boxes are the M-values of a specific gene represented in a row. The background colour visualises the strength of the induction/lower expression (red/green) by the colour intensity. Click here for file

Additional file 6 **Heat map of cluster F of the eight clusters calculated by K-means clustering of the transcriptional data obtained by microarray analysis of the *S. meliloti *1021 pH shock time course experiment.** Cluster F is almost exclusively composed of genes playing a role in chemotaxis and motility. Genes in this cluster showed a progressive permanent repression for the duration of the time course. Each column of the heat map represents one time point after shift from pH 7.0 to pH 5.75 in the following order: 3, 8, 13, 18, 33, and 63 minutes. The values in the boxes are the M-values of a specific gene represented in a row. The background colour visualises the strength of the induction/lower expression (red/green) by the colour intensity. Click here for file

Additional file 7 **Heat map of cluster G of the eight clusters calculated by K-means clustering of the transcriptional data obtained by microarray analysis of the *S. meliloti *1021 pH shock time course experiment.** Cluster G consists of several genes involved in nitrogen uptake and utilization. Genes in this cluster were transiently down-regulated with a minimum before 20 minutes after pH shift. Each column of the heat map represents one time point after shift from pH 7.0 to pH 5.75 in the following order: 3, 8, 13, 18, 33, and 63 minutes. The values in the boxes are the M-values of a specific gene represented in a row. The background colour visualises the strength of the induction/lower expression (red/green) by the colour intensity. Click here for file

Additional file 8 **Heat map of cluster H of the eight clusters calculated by K-means clustering of the transcriptional data obtained by microarray analysis of the *S. meliloti *1021 pH shock time course experiment.** The small cluster H is formed by genes with distinct biological functions and a high variation in their expression levels. Genes in this cluster showed an ultra short transient repression for the first time point 3 minutes after pH shift. Each column of the heat map represents one time point after shift from pH 7.0 to pH 5.75 in the following order: 3, 8, 13, 18, 33, and 63 minutes. The values in the boxes are the M-values of a specific gene represented in a row. The background colour visualises the strength of the induction/lower expression (red/green) by the colour intensity. Click here for file

Additional file 9 **Spreadsheet of the 230 genes used for clustering analysis.** Given is the name of each gene and its corresponding annotation, as well as the M-values calculated for the time course experiment. The last column indicates the cluster, in which the gene was distributed by K-means clustering. Click here for file
