# Supplementary figures and images for: The time course of the transcriptomic response of Sinorhizobium meliloti 1021 following a shift to acidic pH
Source: BMC Microbiol. 2009 Feb 15;9:37. doi: 10.1186/1471-2180-9-37 (PMC2651895; doi:10.1186/1471-2180-9-37)

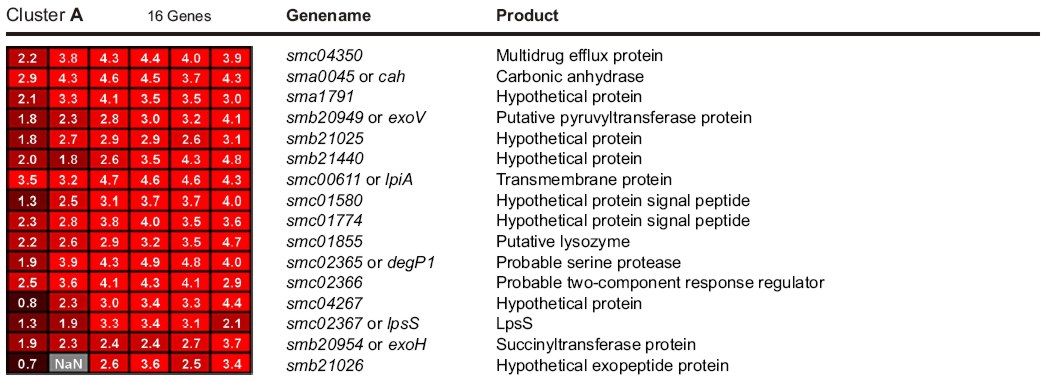

Supplement: Additional file 1 — Heat map of cluster A. By K-means the transcriptional data obtained by microarray analysis of the S. meliloti 1021 pH shock time course experiment were grouped into eight clusters. In cluster A, genes exhibiting a strong and permanent induction were accumulated. Genes in this cluster remained up-regulated for the whole observation period. Presumably, these genes have a special impact for S. meliloti in facing low pH conditions. Each column of the heat map represents one time point after shift from pH 7.0 to pH 5.75 in the following order: 3, 8, 13, 18, 33, and 63 minutes. The values in the boxes are the M-values of a specific gene represented in a row. The background colour visualises the strength of the induction/lower expression (red/green) by the colour intensity. [file 1471-2180-9-37-S1.jpeg]

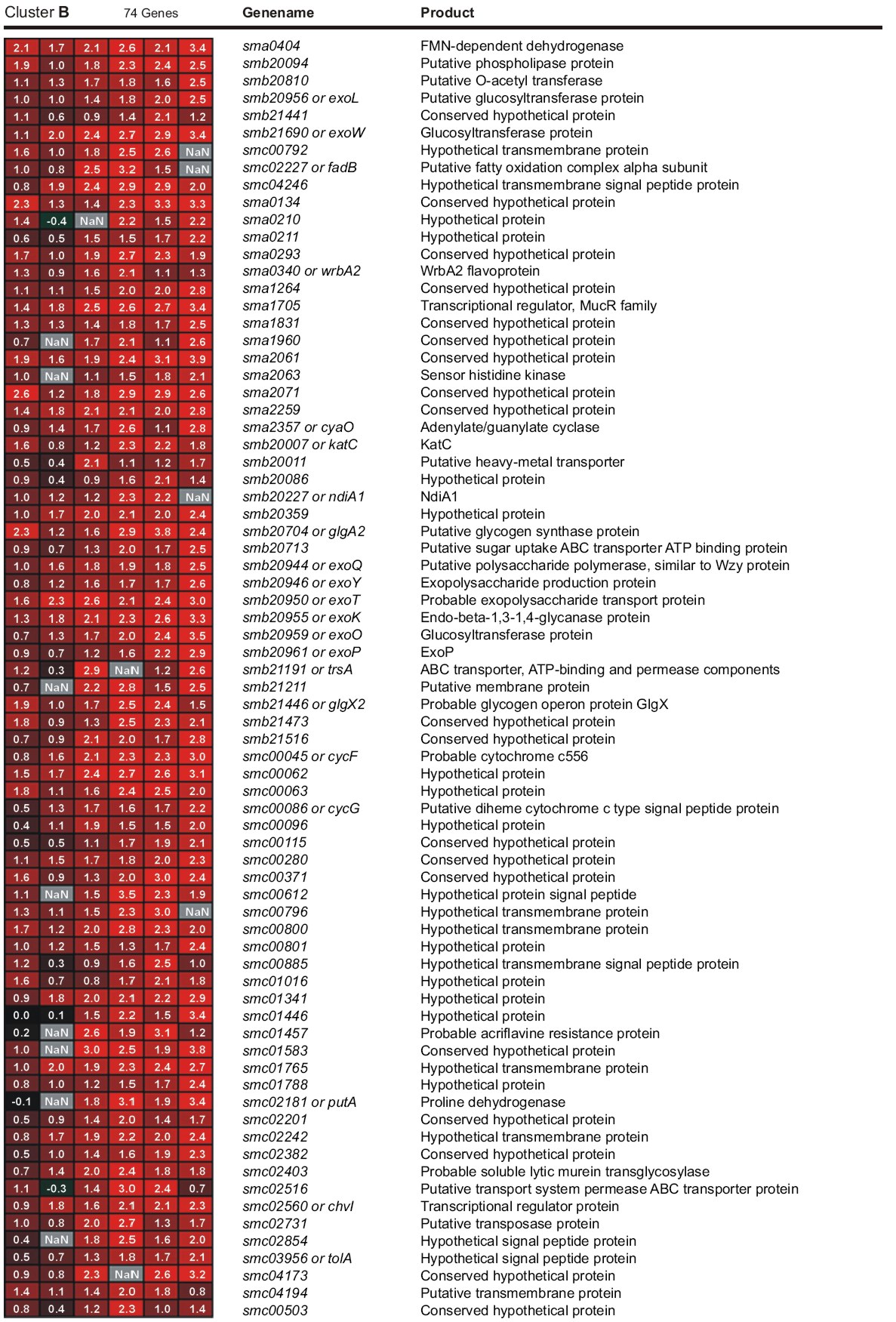

Supplement: Additional file 2 — Heat map of cluster B of the eight clusters calculated by K-means clustering of the transcriptional data obtained by microarray analysis of the S. meliloti 1021 pH shock time course experiment. Cluster B is the largest cluster. The genes in this cluster are permanently up-regulated in response to the pH shift. It contains exo genes responsible for the biosynthesis of succinoglycan and several genes which are rpoE2 dependently regulated. Among the genes in cluster B several encode for hypothetical proteins. Each column of the heat map represents one time point after shift from pH 7.0 to pH 5.75 in the following order: 3, 8, 13, 18, 33, and 63 minutes. The values in the boxes are the M-values of a specific gene represented in a row. The background colour visualises the strength of the induction/lower expression (red/green) by the colour intensity. [file 1471-2180-9-37-S2.jpeg]

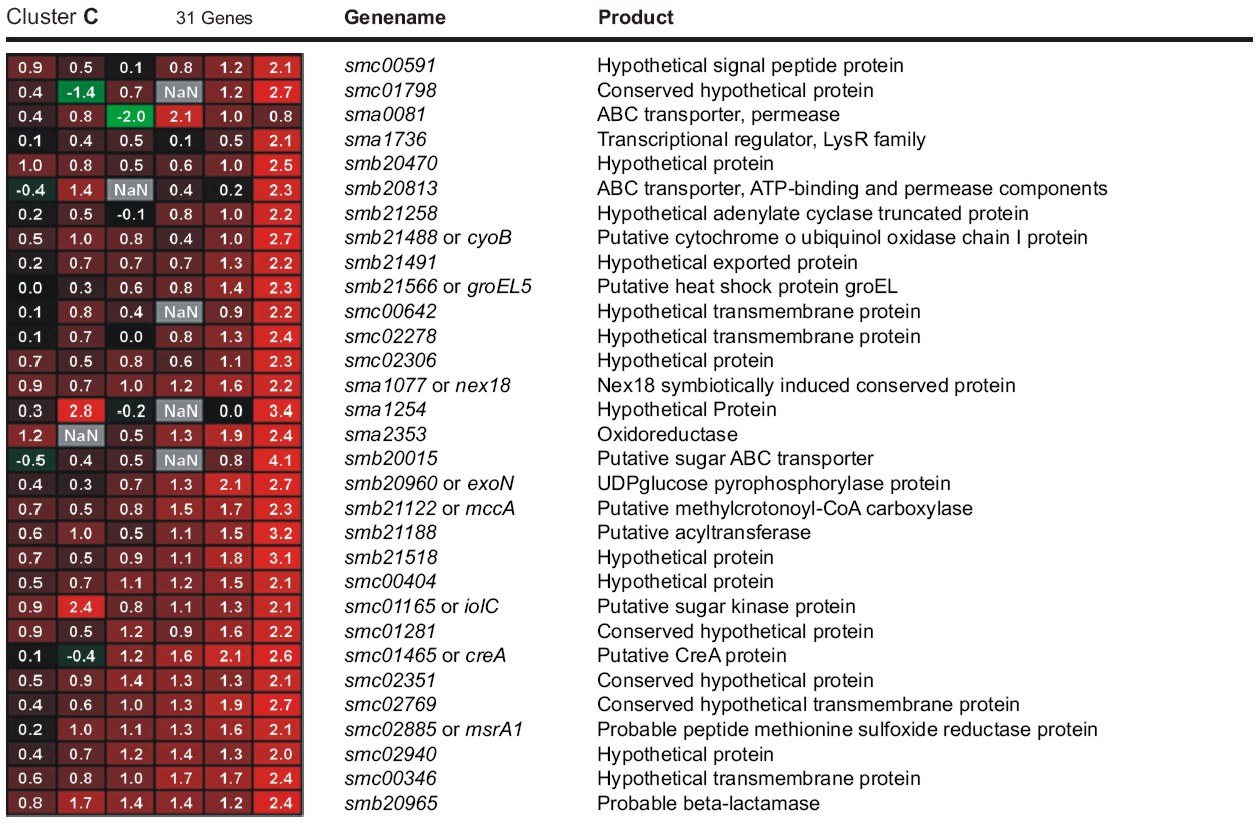

Supplement: Additional file 3 — Heat map of cluster C of the eight clusters calculated by K-means clustering of the transcriptional data obtained by microarray analysis of the S. meliloti 1021 pH shock time course experiment. Cluster C contains over 50% hypothetical genes and a gene coding for a chaperone as well as for a component of a low O2 affinity oxidase. The genes in cluster C showed a progressive permanent induction in their mean expression behaviour. Each column of the heat map represents one time point after shift from pH 7.0 to pH 5.75 in the following order: 3, 8, 13, 18, 33, and 63 minutes. The values in the boxes are the M-values of a specific gene represented in a row. The background colour visualises the strength of the induction/lower expression (red/green) by the colour intensity. [file 1471-2180-9-37-S3.jpeg]

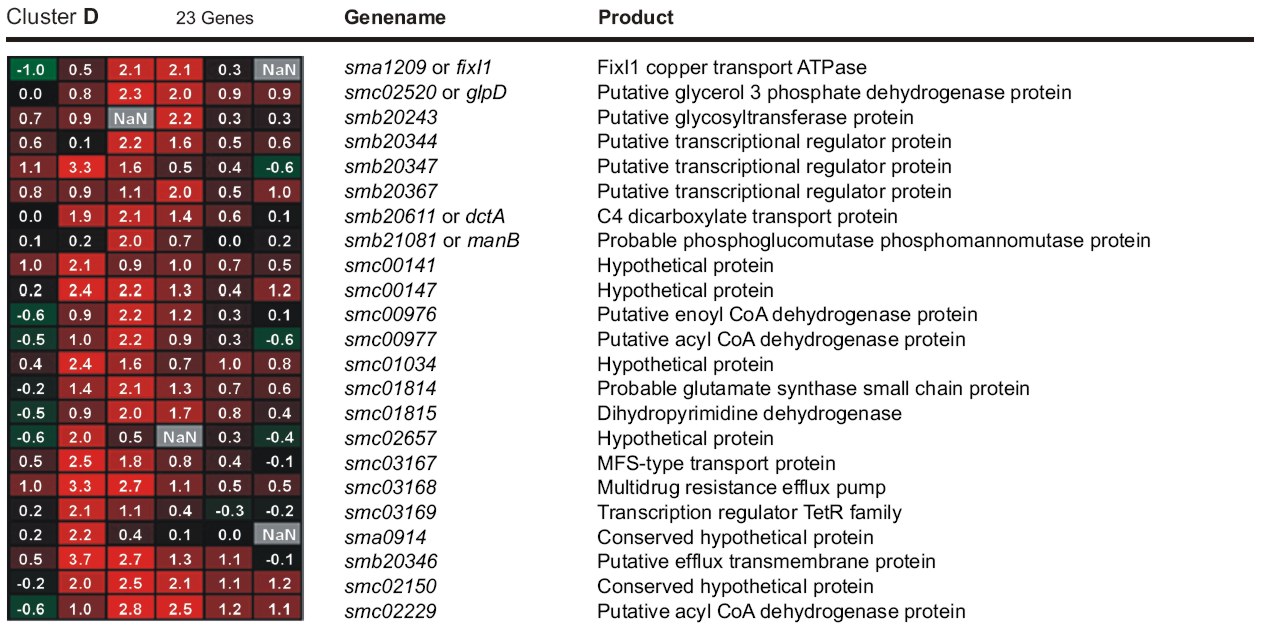

Supplement: Additional file 4 — Heat map of cluster D of the eight clusters calculated by K-means clustering of the transcriptional data obtained by microarray analysis of the S. meliloti 1021 pH shock time course experiment. Cluster D comprises carbon uptake and fatty acid degradation genes. The containing genes were transiently up-regulated during the first 10 to 30 minutes following the pH shift. Each column of the heat map represents one time point after shift from pH 7.0 to pH 5.75 in the following order: 3, 8, 13, 18, 33, and 63 minutes. The values in the boxes are the M-values of a specific gene represented in a row. The background colour visualises the strength of the induction/lower expression (red/green) by the colour intensity. [file 1471-2180-9-37-S4.jpeg]

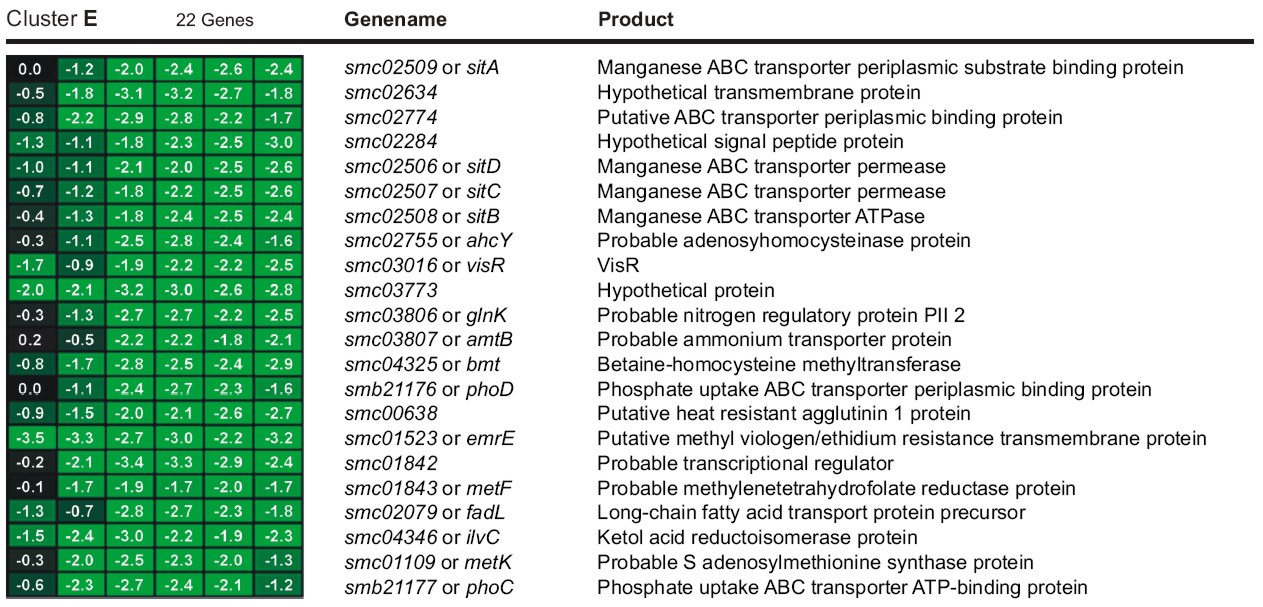

Supplement: Additional file 5 — Heat map of cluster E of the eight clusters calculated by K-means clustering of the transcriptional data obtained by microarray analysis of the S. meliloti 1021 pH shock time course experiment. Cluster E contains genes involved in nitrogen metabolism, ion transport and amino acid biosynthesis. These genes were decreased in their expression value up to 20 minutes after pH shift and then stayed permanently down-regulated. Each column of the heat map represents one time point after shift from pH 7.0 to pH 5.75 in the following order: 3, 8, 13, 18, 33, and 63 minutes. The values in the boxes are the M-values of a specific gene represented in a row. The background colour visualises the strength of the induction/lower expression (red/green) by the colour intensity. [file 1471-2180-9-37-S5.jpeg]

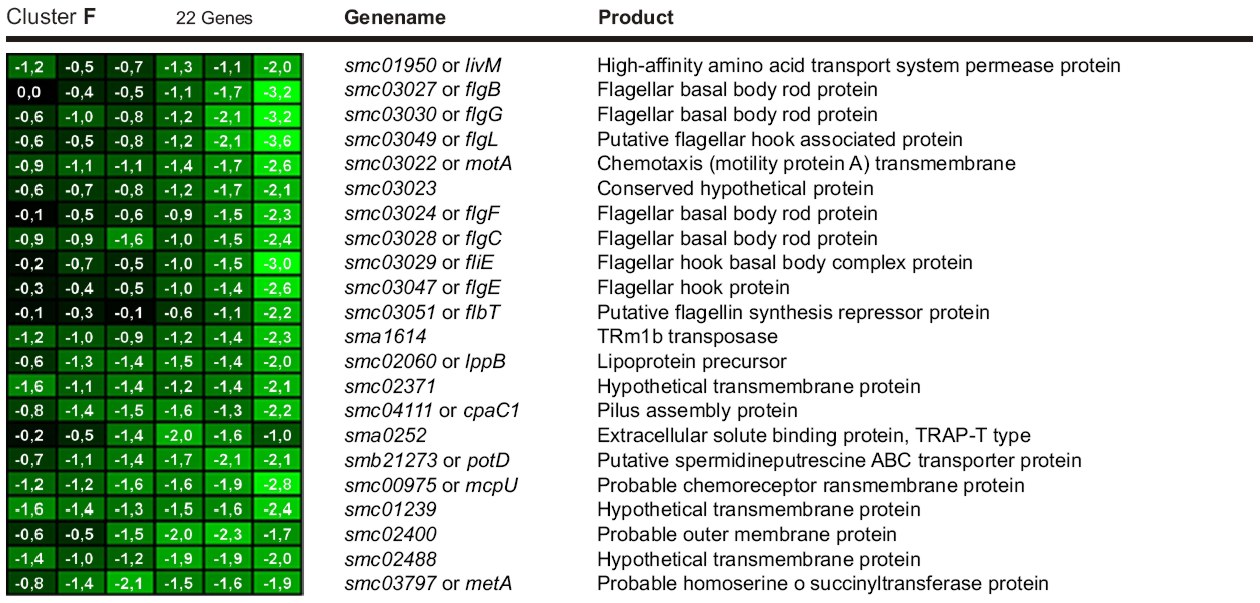

Supplement: Additional file 6 — Heat map of cluster F of the eight clusters calculated by K-means clustering of the transcriptional data obtained by microarray analysis of the S. meliloti 1021 pH shock time course experiment. Cluster F is almost exclusively composed of genes playing a role in chemotaxis and motility. Genes in this cluster showed a progressive permanent repression for the duration of the time course. Each column of the heat map represents one time point after shift from pH 7.0 to pH 5.75 in the following order: 3, 8, 13, 18, 33, and 63 minutes. The values in the boxes are the M-values of a specific gene represented in a row. The background colour visualises the strength of the induction/lower expression (red/green) by the colour intensity. [file 1471-2180-9-37-S6.jpeg]

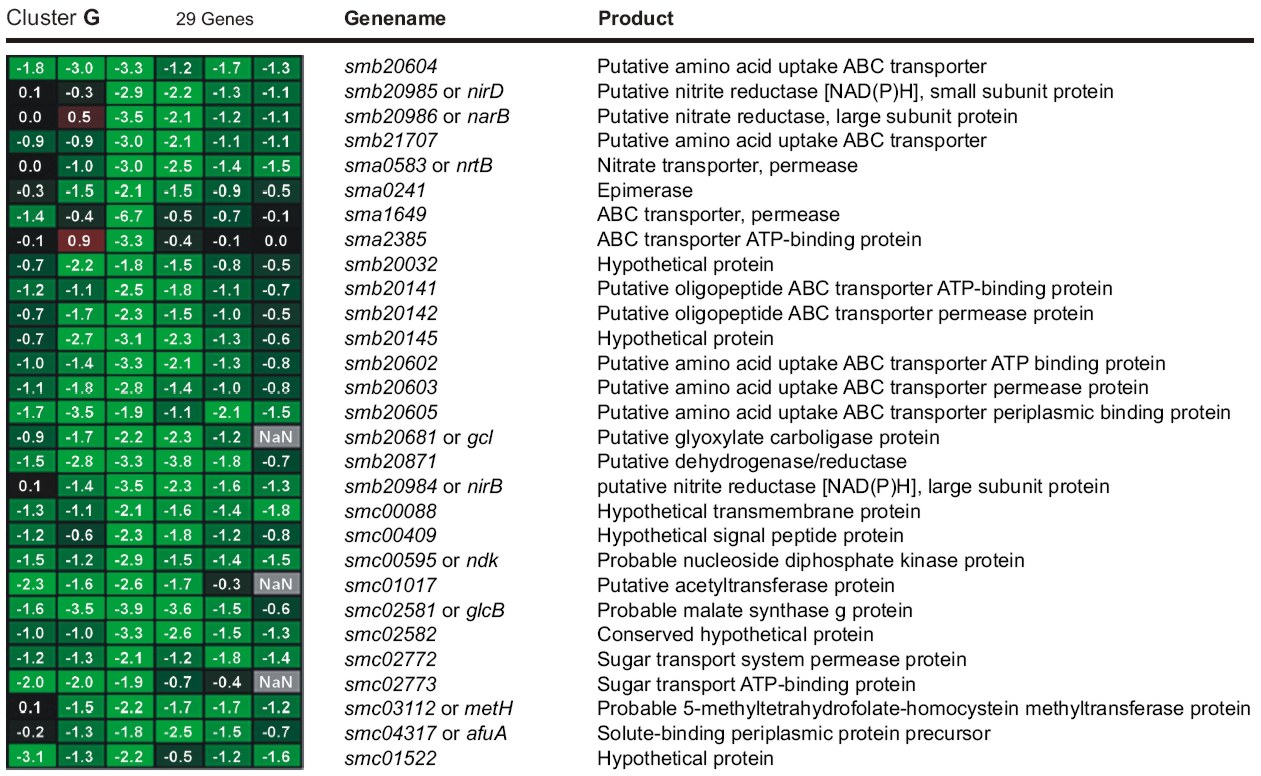

Supplement: Additional file 7 — Heat map of cluster G of the eight clusters calculated by K-means clustering of the transcriptional data obtained by microarray analysis of the S. meliloti 1021 pH shock time course experiment. Cluster G consists of several genes involved in nitrogen uptake and utilization. Genes in this cluster were transiently down-regulated with a minimum before 20 minutes after pH shift. Each column of the heat map represents one time point after shift from pH 7.0 to pH 5.75 in the following order: 3, 8, 13, 18, 33, and 63 minutes. The values in the boxes are the M-values of a specific gene represented in a row. The background colour visualises the strength of the induction/lower expression (red/green) by the colour intensity. [file 1471-2180-9-37-S7.jpeg]

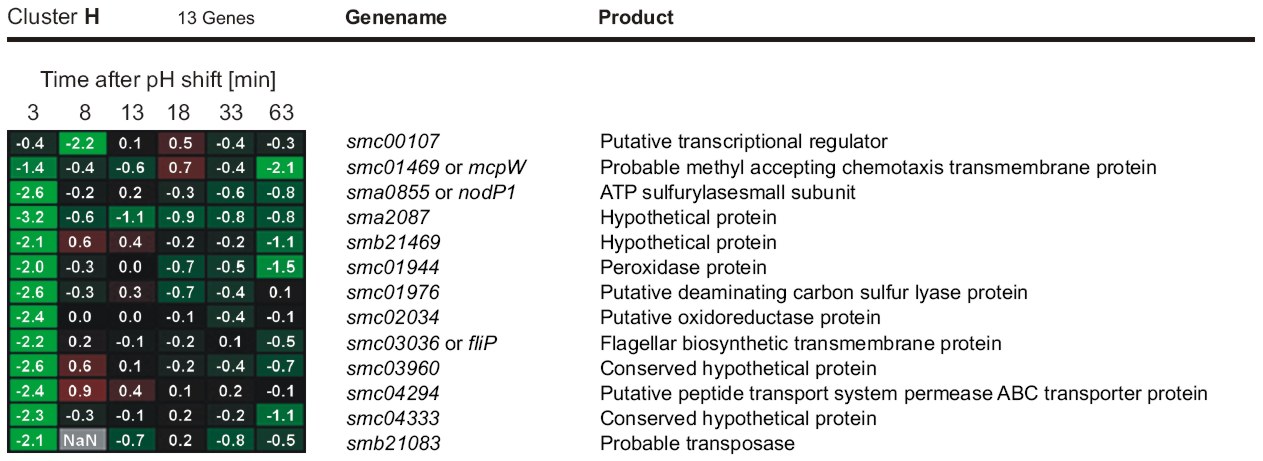

Supplement: Additional file 8 — Heat map of cluster H of the eight clusters calculated by K-means clustering of the transcriptional data obtained by microarray analysis of the S. meliloti 1021 pH shock time course experiment. The small cluster H is formed by genes with distinct biological functions and a high variation in their expression levels. Genes in this cluster showed an ultra short transient repression for the first time point 3 minutes after pH shift. Each column of the heat map represents one time point after shift from pH 7.0 to pH 5.75 in the following order: 3, 8, 13, 18, 33, and 63 minutes. The values in the boxes are the M-values of a specific gene represented in a row. The background colour visualises the strength of the induction/lower expression (red/green) by the colour intensity. [file 1471-2180-9-37-S8.jpeg]
